# In Vitro Anti-Inflammatory Study of Limonoids Isolated from *Chisocheton* Plants

**DOI:** 10.3390/cimb46010058

**Published:** 2024-01-20

**Authors:** Erina Hilmayanti, Xuhao Huang, Supriatno Salam, Nurlelasari, Unang Supratman, Kazuya Kabayama, Koichi Fukase

**Affiliations:** 1 Department of Chemistry, Graduate School of Science, Osaka University, Toyonaka 560-0043, Japan; 2 Department of Chemistry, Faculty of Mathematics and Natural Sciences, Universitas Padjadjaran, Jatinangor 45363, Indonesia; 3 Faculty of Pharmacy, Universitas Mulawarman, Samarinda 75123, Indonesia; 4 Central Laboratory, Universitas Padjadjaran, Jatinangor 45363, Indonesia; 5 Project Research Center for Fundamental Sciences, Osaka University, Toyonaka 560-0043, Japan

**Keywords:** limonoid, *Chisocheton*, anti-inflammatory, Meliaceae

## Abstract

*Chisocheton* plants from the family Meliaceae have traditionally been used to treat several diseases; however, scientific evidence is limited. The most abundant chemical constituents of this plant are the limonoids, which are known for their various biological activities, including anti-inflammatory effects. However, the anti-inflammatory effects and underlying mechanisms of action of the constituents of *Chisocheton* plants have not been fully explored. In this report, we evaluated the anti-inflammatory activity of 17 limonoid compounds from *Chisocheton* plant primarily by measuring their inhibitory effects on the production of pro-inflammatory cytokines, including TNF-α, IL-6, IL-1β, and MCP-1, in LPS-stimulated THP-1 cells using an ELISA assay. Compounds **3**, **5**, **9**, and **14**–**17** exhibited significant activity in inhibiting the evaluated pro-inflammatory markers, with IC_50_ values less than 20 µM and a high selectivity index (SI) range. Compounds **3**, **5**, **9**, and **15** significantly suppressed the expression of phosphorylated p38 MAPK in THP-1 cells stimulated with LPS. These findings support the use of limonoids from *Chisocheton* plants as promising candidates for anti-inflammatory therapy.

## 1. Introduction

Inflammation is a natural protective response against pathogens such as viruses, bacteria, harmful substances, injury, and irradiation. This occurs in an attempt to eliminate the stimuli and initiate the healing process that involves immune responses [1]. In the immune responses, monocytes and macrophages play a pivotal role in inflammatory processes due to their ability to synthesize and secrete pro- and anti-inflammatory cytokines. THP-1 cell is a human monocytic cell line that can be differentiated to a macrophage-like state to modulate monocyte and macrophage activities [2]. However, excessive inflammatory responses can lead to various acute and chronic inflammation-related diseases. Inflammatory stimuli, such as lipopolysaccharides (LPS) derived from Gram-negative bacteria, are commonly known as endotoxins and act as pathogen-associated molecular patterns (PAMPs), serving as potent Toll-like receptor 4 (TLR4) agonists. TLR4 is highly responsive to LPS and undergoes complex downstream molecular recognition. LPS binds to cluster differentiation-14 (CD14) and myeloid differentiation factor-2 (MD-2), and associates with TLR4 to activate this pathway. Upon activation of the TLR4/MD-2 complex, the production of inflammatory cytokines such as TNF-α, IL-1, and IL-6 is initiated. In the absence of the agonist, the monomeric and dimeric forms of the TLR4/MD-2 complex are in equilibrium [3,4,5].

Historically, natural products have played a pivotal role in drug discovery and are considered rich sources of molecules with therapeutic effects. Over 60% of the commercially used medicines are either natural products or their derivatives [6,7]. Limonoids are natural products that exhibit various bioactivities, including antibacterial [8], antifungal [9], insect antifeedant [10], insecticidal [11], antimalarial [12], antioxidant [13], cytotoxic [14], anticancer [15], antiviral [16], and anti-inflammatory [17] properties.

**Figure 1:**
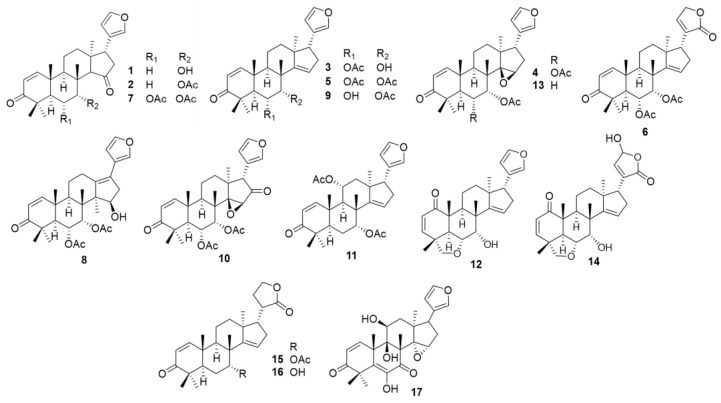
Structure of compounds **1**–**17**.

Borges et al. (2016) reported that gedunin, a limonoid from the Meliaceae family, exhibited immunomodulatory effect and multitarget properties in vitro and in vivo. The compound has considerable capability to modulate the activation of anti-inflammatory factors and reduce pro-inflammatory mediators triggered by LPS, a TLR ligand. This result suggests the potential of the limonoid compound as an anti-inflammatory and immunomodulatory agent to enhance the treatment of inflammation caused by bacterial pathogens [18]. *Chisocheton*, a genus from the Meliaceae family, is known as one of the sources of limonoid compounds. In our previous study, we isolated 17 limonoids, namely 7α-hydroxyneotrichilenone (**1**), 7α-acetylneotrichilenone (**2**), 6α-O-acetyl-7-deacetylnimocinol (**3**), 6α-(acetoxy)-14β,15β-epoxyazadirone (**4**), dysobinin (**5**), lasiocarpine A (**6**), toonaciliatone F (**7**), toonaciliatone C (**8**), nimonol (**9**), 6α-acetylepoxyazadirone (**10**), 11α-acetoxyazadirone (**11**), ceramicine B (**12**), trichilenone acetate (**13**), pentandricine (**14**), pentandricine B (**15**), pentandricine C (**16**), and pentandricine G (**17**) (Figure 1) from Indonesian *Chisocheton* plant and investigated their cytotoxicity against several cancer cell lines [19,20,21,22,23,24]. Inflammation has been associated with cancer, and anti-inflammatory therapy has been reported to be effective in managing the initial stages of neoplastic progression and conversion to malignancy by reducing tumor-promoting properties, such as pro-inflammatory cytokines [25]. Therefore, we evaluated the anti-inflammatory potential of limonoids in vitro by targeting the inhibition of pro-inflammatory markers.

## 2. Materials and Methods

### 2.1. Chemicals and Reagents

Our previous studies have reported the isolation and characterization of the 17 limonoids evaluated in this report: 7α-hydroxyneotrichilenone (**1**), 7α-acetylneotrichilenone (**2**), 6α-O-acetyl-7-deacetylnimocinol (**3**), 6α-(acetoxy)-14β,15β-epoxyazadirone (**4**), dysobinin (**5**), lasiocarpine A (**6**), toonaciliatone F (**7**), and toonaciliatone C (**8**) were isolated from the fruit of *C. lasiocarpus* [19,20]; nimonol (**9**), and 6α-acetylepoxyazadirone (**10**) were isolated from seeds of *C. macrophyllus* [18]; and 11α-acetoxyazadirone (**11**), ceramicine B (**12**), trichilenone acetate (**13**), pentandricine (**14**), pentandricine B (**15**), pentandricine C (**16**), and pentandricine G (**17**) were isolated from the stembark of *C. pentandrus* [19,20,21,22,23,24]. All compounds were diluted in ethanol (99.5%) that was purchased from Fujifilm Wako Pure Chemical Co., Ltd. (Osaka, Japan).

Fetal bovine serum (FBS; 10270-106) was obtained from Thermo Fisher Scientific (Waltham, MA, USA). High-glucose Dulbecco’s modified eagle medium (D-MEM) (043-30085), Roswell Park Memorial Institute (RPMI)-1640 (189-02025), penicillin–streptomycin (x100; 168-23191), *Escherichia coli* lipopolysaccharide (LPS,125-05181), dexamethasone (047-18863), phosphate buffer saline (−) (PBS, 164-28713), bovine serum albumin (BSA, 019-15101), and globulin free were obtained from Fujifilm Wako Pure Chemical Co., Ltd. (Osaka, Japan). Phorbol 12-myristate 13-acetate (PMA) was purchased from Focus Biomolecules (Plymouth Meeting, PA, USA), while a Cell Counting Kit-8 (CCK-8; 343-07623) was purchased from Dojindo Molecular Technology (Kumamoto, Japan). P-nitrophenyl phosphate (PNPP) (49889-19) was purchased from Molecular Technology (Tokyo, Japan), and Human ELISA kits for human TNF-α (88-7346-88), human IL-6 (88-7066-88), human IL-1β (88-7261-88), and human MCP-1 (88-7399-88) were purchased from Invitrogen ThermoFisher Scientific (Carlsbad, CA, USA). Anti-p38 MAPK (9212) and anti-phospho-p38 MAPK (Thr180/Tyr182; 9211) antibodies were purchased from Cell Signaling Technology (Danvers, MA, USA). Rabbit anti-IgG (H + L-chain) (Rabbit) pAb-HRP (458) was purchased from MBL (Nagoya, Japan). Anti-β-actin (A5441) and Immobilon Western Chemiluminescent HRP Substrate (WBKLS0100) were from Sigma-Aldrich (St. Louis, MO, USA).

### 2.2. Cell Culture

THP-1 cells (JCRB0112) were purchased from the JCRB Cell Bank (Ibaraki, Osaka, Japan) and cultured in RPMI-1640. HEK-Blue^TM^ hTLR4 cells were purchased from Invivo Gen (San Diego, CA, USA) and were cultured in high-glucose D-MEM. Both media were supplemented with 10% FBS and 1% penicillin–streptomycin. All cells were incubated at 37 °C in a 5% carbon dioxide atmosphere.

### 2.3. SEAP Reporter Gene Assay

The SEAP Reporter Gene Assay was conducted as previously described [26]. HEK-Blue^TM^ hTLR4 cells (2 × 10^4^ cells/well) were seeded in 96-well plates and were serum-starved for 24 h at 37 °C in a 5% carbon dioxide atmosphere. After incubation, the culture medium was aspirated, and the cells were washed with saline. High-glucose D-MEM supplemented with 0.1% FBS and 1% penicillin-streptomycin was added and treated with or without the 17 limonoids at a concentration of 20 µM for 1 h, followed by LPS addition at a concentration of 10 ng/mL for the LPS-stimulated group. After incubation at 37 °C for 18 h, the supernatants were collected and PNPP in PBS solution (0.8 mM, 100 μL) was added, followed by 4 h of shading at 25 °C. The optical density (OD) of the samples was measured at 405 nm using a microplate reader (BioTek Cytation 5 Cell Imaging Multimode Reader, Agilent Technologies, Santa Clara, CA, USA).

### 2.4. Effects of 17 Tested Compounds on LPS-Induced Pro-Inflammatory Cytokine Production by ELISA Assay

The logarithmic growth phase of THP-1 cells was achieved by seeding in 96-well plates at a density of 5 × 10^4^ cells/well and differentiated for 24 h in the presence of PMA (0.5 μM solution in medium). After 24 h of incubation, the culture medium was removed from the 96-well plate and the cells were washed with PBS. Fresh RPMI-1640 medium supplemented with 0.1% FBS and 1% Penicillin–Streptomycin was added to each well and incubated at 37 °C overnight. The cells were pretreated with 17 limonoids at the corresponding concentration for 1 h, followed by the addition of LPS (10 ng/mL) and then incubation at 37 °C for 18 h. Finally, 50 μL of each culture supernatant solution was taken to determine the concentrations of IL-6, MCP-1, and IL-1β using the corresponding commercially available human ELISA kits following the manufacturer’s instructions. The analyte concentration in the supernatant was measured in pg/mL, and the inhibition level was expressed as a fold change compared to the LPS (−) control group. To determine the IC_50_ value, the dose-response data were normalized to the LPS-stimulated-only group, and the percentage of inhibition was calculated using the following formula:

$$\%\;Inhibition=\frac{\left(Absorbance\;of\;LPS\left(+\right)\;control-Absorbance\;of\;compound\;treatment\;with\;LPS\right)}{\left(Absorbance\;of\;LPS\left(+\right)\;control-Absorbance\;of\;LPS\left(-\right)\;control\right)}\times 100$$

Furthermore, the % inhibition data were interpolated into linear equations to determine the IC_50_ values.

### 2.5. Effects of 17 Limonoids on the Viability of HEK-Blue^TM^ hTLR4 and THP-1 Cells

The viability of HEK-Blue^TM^ hTLR4, RAW 274.6, and THP-1 cells was evaluated using the Dojindo Cell Counting Kit-8 (CCK-8) according to the manufacturer’s instructions. The viability of HEK-Blue^TM^ hTLR4 and THP-1 cells was assessed under identical conditions to the corresponding assay treatments. HEK-Blue^TM^ hTLR4 cells (2 × 10^4^ cells/well) were seeded in 96-well plates and serum-starved for 24 h at 37 °C in a 5% CO_2_ atmosphere. After incubation, the culture medium was aspirated, and the cells were washed with saline. High-glucose D-MEM supplemented with 0.1% FBS and 1% penicillin–streptomycin was added and pretreated with or without the 17 limonoids at a concentration of 20 µM for 1 h, followed by LPS addition at a concentration of 10 ng/mL. After 18 h of incubation, a 10% (*v*/*v*) CCK-8 solution was added to each well and incubated at 37 °C for 1 h, and cell viability was calculated. 

The THP-1 cells at 5 × 10^4^ cells/well were differentiated for 24 h in the presence of PMA (0.5 μM solution in medium). After 24 h of incubation, the culture medium was removed from the 96-well plate and the cells were washed with PBS. Fresh RPMI-1640 medium supplemented with 0.1% FBS and 1% penicillin–streptomycin was added to each well and incubated at 37 °C for overnight. The cells were pretreated with 17 limonoids at the corresponding concentration for 1 h followed by the addition of LPS (10 ng/mL) and incubated at 37 °C for 18 h. Furthermore, CCK-8 solution was added to each well with a final concentration was 10% (*v*/*v*) and incubated at 37 °C. After 1 h, the absorbance of each well was measured at 405 nm by using a microplate reader (BioTek Cytation 5 Cell Imaging Multimode Reader). Cell viability was calculated using the following formula:

$$\%\;Cell\;viability=\frac{\left(Absorbance\;of\;sample-Absorbance\;of\;Background\right)}{\left(Absorbance\;of\;control-Absorbance\;of\;Background\right)}\times 100$$

where the absorbance of the sample is the absorbance of cells treated by the compound with or without LPS, the absorbance of the control is the absorbance of LPS (−)-treated cells, and the background absorbance is the absorbance of a well containing only the medium. The results were expressed as 100% cell viability.

### 2.6. Effects of Compounds ***3***, ***5***, ***9***, and ***15*** on LPS-Stimulated MAPK Activation by Western Blotting

THP-1 cells were seeded at a density of 1 × 10^6^ cells/well in 24-well plates. The cells were then pretreated with a concentration of 10 µM of compounds **3**, **5**, **9**, or **15** for 1 h. Subsequently, LPS (10 ng/mL) was added to the cells, which were incubated for 18 h. The protein concentrations were determined using a bicinchoninic acid (BCA) assay kit.

For Western blot analysis, proteins were separated by SDS-PAGE and transferred onto a polyvinylidene fluoride (PVDF) membrane. The membranes were blocked with 2.5% BSA in Tris-buffered saline-Tween (TBST) for 1 h at room temperature. After three washes with TBST, the membranes were incubated with a diluted primary antibody solution (anti-phosphorylated-p38 MAPK or anti-p38 MAPK) at a 1:1000 dilution for 1 h at room temperature. After three washes with TBS-T, the membranes were incubated with a secondary antibody conjugated to horseradish peroxidase (HRP; 1:4000 dilution) for 30 min at room temperature and subsequently detected by chemiluminescence. Finally, the bands were visualized and quantified using an ImageQuant 800 GxP system (Cytiva, MA, USA). The results were expressed as ratios relative to the reference protein β-actin.

### 2.7. Statistical Analysis

All experiments were performed in quadruplicate and the results are expressed as mean ± standard deviation (SD). Multiple datasets were compared using one-way analysis of variance (ANOVA), followed by Dunnett’s multiple comparisons test which was performed using GraphPad Prism 10.1.0. Statistical significance was set at *p* < 0.05.

## 3. Results

### 3.1. Inhibition of SEAP Release in LPS-Induced HEK-Blue^TM^ hTLR4 Cells

**Figure 2:**
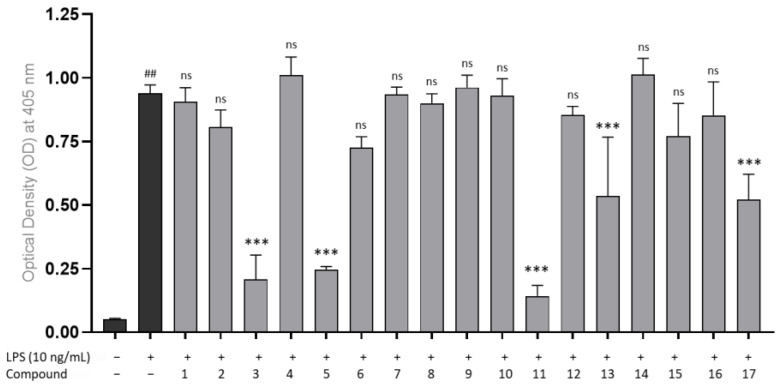
Antagonist effects of LPS-stimulated TLR4-dependent NF-kB activation in HEK-Blue^TM^ hTLR4 cells by compounds (**1**–**17**) at a concentration of 20 µM, and LPS-stimulated (10 ng/mL) after 1 h. All data from four independent experiments are expressed as mean ± SD. ## *p* < 0.0001 vs. culture medium-only control group; ns: not significant, *p* > 0.1, *** *p* < 0.0001, vs. LPS stimulated-only group. The *p*-value was analyzed by one-way ANOVA, followed by Dunnett’s multiple comparisons test which was performed using GraphPad Prism 10.1.0.

The evaluation of nuclear factor kappa-light-chain-enhancer of activated B cells (NF-κB) activation with and without LPS-induction against HEK-Blue^TM^ hTLR4 cells was performed using a secreted embryonic alkaline phosphatase (SEAP) reporter gene assay to determine the effect of compounds **1**–**17** on NF-κB activation via TLR4. As shown in Figure 2, compounds **3**, **5**, **11**, **13**, and **17** significantly inhibited SEAP release, which correlated with NF-κB activity inhibition (*p* < 0.01).

### 3.2. Inhibition of the Release of TNF-α, IL-6, IL-1β, and MCP-1 in LPS-Stimulated THP-1 Cells

**Figure 3:**
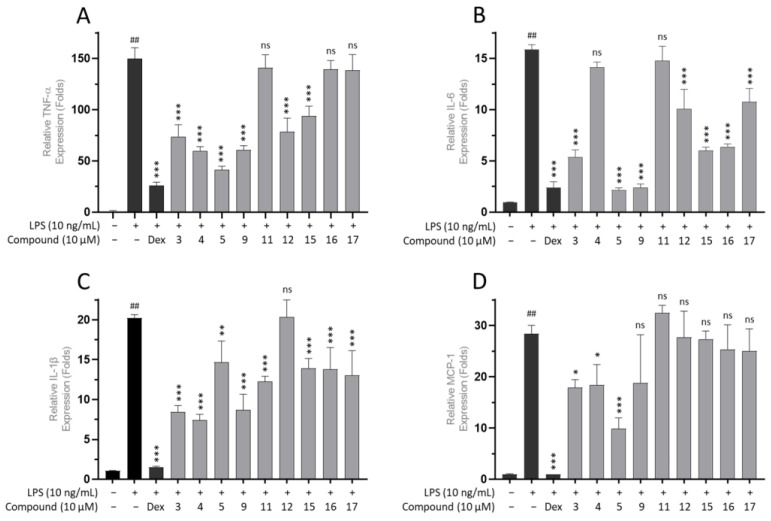
Relative effects of compounds **3**–**5**, **9**, **11**, **12**, **15**–**17,** and dexamethasone (DEX) as positive control at concentrations of 10 µM on the production of pro-inflammatory markers; TNF-α (**A**), IL-6 (**B**), IL-1β (**C**), and MCP-1 (**D**) in LPS-stimulated THP-1 cells. Data were normalized to the negative control group. All data from four independent experiments are expressed as mean ± SD. ## *p* < 0.0001 vs. LPS (−) control group; ns: not significant, *p* > 0.05, * *p* < 0.01, ** *p* < 0.001, *** *p* < 0.0001 vs. LPS (+) control group. The *p*-value was analyzed using one-way ANOVA, followed by Dunnett’s multiple comparisons test which was performed using GraphPad Prism 10.1.0.

An enzyme-linked immunosorbent assay (ELISA) was performed to determine whether compounds **1**–**17** affected the secretion of pro-inflammatory cytokines, including TNF-α, IL-6, IL-1β, and MCP-1 in LPS-induced THP-1 cells. As depicted in Figure 3, the levels of TNF-α, IL-6, IL-1β, and MCP-1 significantly increased for the LPS (+) (10 ng/mL) treatment group compared to the LPS (−) control group (## *p* < 0.0001). In contrast, treatment with compounds **3**, **5**, and **9** at a concentration of 10 µM significantly reduced the TNF-α, IL-6, IL-1β, and MCP-1 levels compared to the LPS (+)-stimulated group (*p* < 0.01). 

**Figure 4:**
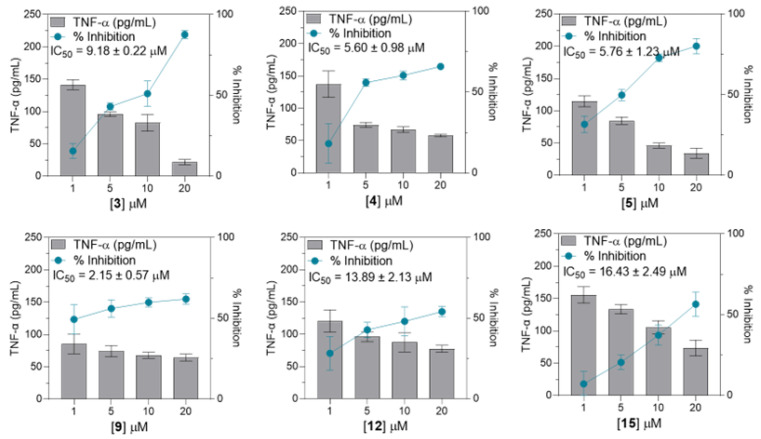
Dose–response graph for compounds **3**, **4**, **5**, **9**, **12**, and **15** to evaluate TNF-α inhibition. All data from four independent experiments are expressed as mean ± SD. The dose–response data were normalized to the LPS-stimulated-only group and converted to % inhibition and interpolated linear equations to determine the IC_50_ values. Data points represent the mean of percentage ± SEM of four independent experiments.

**Figure 5:**
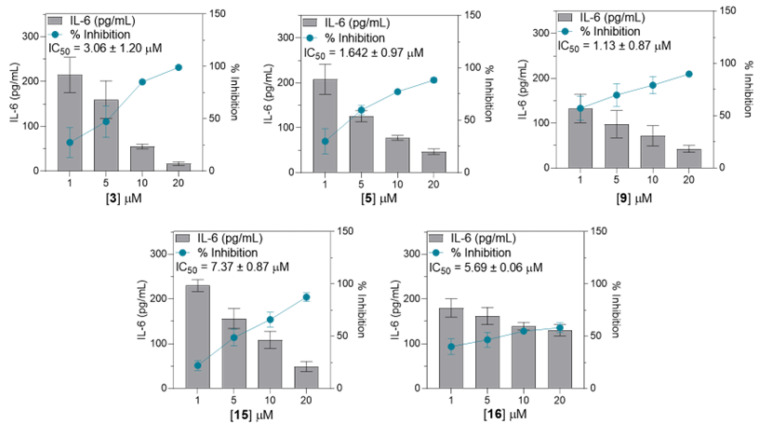
Dose–response graph for compounds **3**, **5**, **9**, **15**, and **16** for the evaluation of IL-6 inhibi-tion. All data from four independent experiments are expressed as mean ± SD. The dose–response data were normalized to the LPS-stimulated-only group, converted to % inhibition, and interpo-lated to linear equations to determine IC_50_ values. Data points represent the mean of percentage ± SEM of four independent experiments.

**Figure 6:**
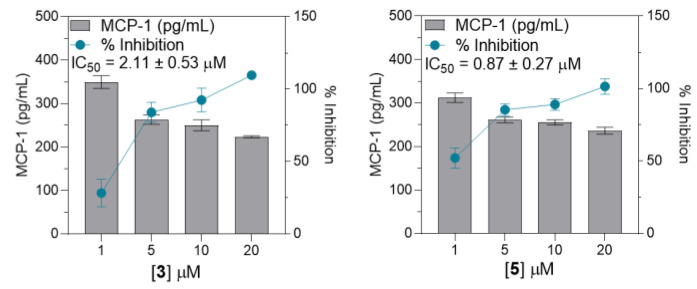
Dose–response graph for compounds **3** and **5** for the evaluation of MCP-1 inhibition. All data from four independent experiments are expressed as mean ± SD. The dose–response data were normalized to the LPS-stimulated-only group, converted to % inhibition, and interpolated to linear equations to determine IC_50_ values. Data points represent the mean of percentage ± SEM of four independent experiments.

Subsequently, compounds showing notable inhibition rates compared to the LPS (+) treatment group (***, *p* < 0.0001), specifically **3**–**5**, **9**, **12**, and **15**, underwent further evaluation using the same method to determine the TNF-α expression levels at concentrations of 1, 5, 10, and 20 µM. For IL-6 expression, compounds **3**, **5**, **9**, **15**, and **16** were further evaluated. In addition, compounds **3** and **5** were evaluated for their effects on MCP-1 expression. Consequently, these seven substances showed concentration-dependent suppression of each corresponding pro-inflammatory signal. The half-maximal inhibitory concentration (IC_50_) ranged from 2.15 to 16.43 µM for TNF-α, 1.13 to 7.37 µM for IL-6, and 0.87 to 2.11 µM for MCP-1 (Shown at Figure 4, Figure 5 and Figure 6). Collectively, compound **9** exhibited the strongest inhibition activity, with IC_50_ values below 3 µM for both TNF-α and IL-6.

Based on the above results, four limonoid compounds, namely **3**, **5**, **9**, and **15** were identified as bioactive phytochemicals that contribute to anti-inflammatory activity. These compounds effectively reduced the production of pro-inflammatory markers, including TNF-α, IL-6, IL-1β, and MCP-1, in LPS-stimulated THP-1 cells, with overall IC_50_ values below 20 µM. Therefore, these compounds were selected for further investigations to explore their anti-inflammatory mechanisms.

### 3.3. Effects of the 17 Tested Compounds on the Cell Viability

The cell viability of HEK-Blue^TM^ hTLR4 and THP-1 cells after 18 h of treatment with the 17 limonoids in the presence or absence of LPS was evaluated using the Cell Counting Kit-8 (CCK-8) method. As shown in Table 1, compounds **2**, **3**, **5**–**9**, **11**, **15**, and **17** had cytotoxic effects on HEK-Blue^TM^ hTLR4 cells at a concentration of 20 µM. Table S1 shows the evaluation of the cytotoxic effects against RAW264.7 macrophage and several human cells. However, except for compounds **5** and **9**, the viability of THP-1 cells was maintained after treatment at a concentration of 10 M. Figure S2 displays the cell viability at various concentrations. The selectivity index (SI) of the selected compounds was calculated; Table 2 summarizes the results.

### 3.4. p-p38 MAPK Signaling Pathway Inhibition in LPS-Stimulated THP-1 Cell by Compounds ***3***, ***5***, ***9***, and ***15***

**Figure 7:**
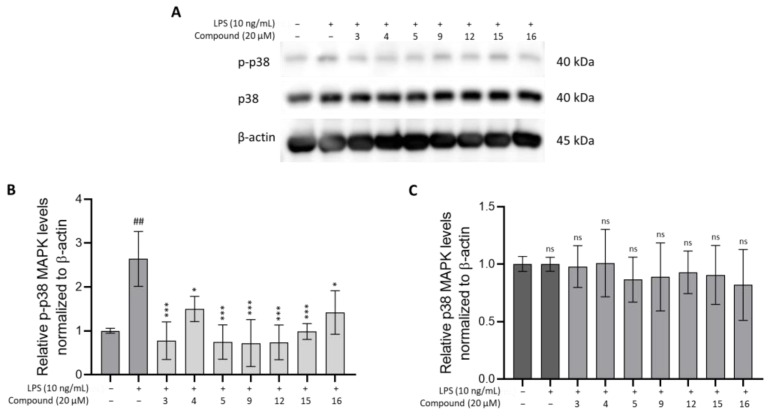
Effects of compounds **3**, **4**, **5**, **9**, **12**, **15**, and **16** on the expression of p-p38 MAPK activation ((**A**) Western blot analysis; (**B**) quantified relative phosphorylated-p38 MAPK; (**C**) quantified relative total p38 MAPK) in LPS-stimulated THP-1 cells. All data from three independent experiments are expressed as mean ± SD. ## *p* < 0.1 vs. culture medium-only control group; ns: not significant; * *p* < 0.01, *** *p* ≤ 0.0001 vs. LPS-only group. The *p*-value was analyzed using one-way ANOVA, followed by Dunnett’s multiple comparisons test which was performed using GraphPad Prism 10.1.0.

To investigate the anti-inflammatory mechanism of compounds **3**, **5**, **9**, and **15**, the protein levels of phosphorylated-p38 MAPK and total p38 MAPK were analyzed using Western blotting. As illustrated in Figure 7, the phosphorylated-p38 MAPK protein ex-pression increased in LPS (+)-stimulated THP-1 cells compared to that in LPS (−)-treated cells. However, treatment with compounds **3**, **5**, **9**, and **15** at a concentration of 10 µM significantly suppressed the p-p38 MAPK levels compared to the LPS (+) group (*p* < 0.01).

Based on these results, compounds **3**, **5**, **9**, and **15** were identified as the most potent anti-inflammatory constituents capable of suppressing the p38 MAPK signaling path-way, which is involved in reducing pro-inflammatory cytokine production in LPS-stimulated THP-1 cells.

## 4. Discussion

*Chisocheton* plants are traditionally used to treat several diseases, such as fever, rheumatism, stomach and kidney complaints, and malaria [27,28]. However, these properties have not yet been validated scientifically. Limonoids are the predominant phytochemical constituents found in the *Chisocheton* plant. Despite the identification of various limonoids with anti-inflammatory properties, the exploration of the anti-inflammatory activity and underlying mechanisms of action of compounds derived from *Chisocheton* plants have not been thoroughly investigated [29].

Limonoids exhibit diverse biological activities. In our previous studies, we isolated and elucidated the compounds. In the present study, we investigate their potential as anti-inflammatory compounds. Compounds **1**–**8** were isolated from the fruit of *C. lasiocarpus* [20,21], **9** and **10** were isolated from the seeds of *C. macrophyllus* [19], and **11**–**17** were isolated from the stem bark of *C. pentandrus* [22,23,24]. These compounds have been reported to have various biological activities, including cytotoxicity against several cell lines such as murine leukemia P-388 [19], breast cancer MCF-7 [21], SMMC7721 human hepatocellular carcinoma, HL-60 human myeloid leukemia, SW480 colon cancer, and A549 lung cancer [30]. They also exhibit neuroprotective [31], anti-inflammatory related to nitric oxide (NO) production, anti-multidrug resistance [32], antiviral [33], insecticidal [34,35], antifungal [36], anti-lipid droplet accumulation [37] [35], and anti-plasmodial [38] properties. These reports indicate the broad spectrum of biological activities of limonoids.

Sun et al. (2022) reported on anti-inflammatory limonoid compounds from *Munronia* plants that exhibited strong inhibition of nitric oxide (NO) production in LPS-stimulated RAW264.7 macrophages. Notably, the ring-intact-type limonoid compound exhibited the most significant activity, which further demonstrated its inhibition of the initiation and assembly of NLRP3 inflammasome [39]. In this study, we evaluated the anti-inflammatory activities of 17 ring-intact type limonoids isolated from *Chisocheton* plants. 

Initial screening for anti-inflammatory compounds was based on NF-κB activation, and this activation can be characterized by the secretion of SEAP. NF-κB plays a pivotal role in the inflammatory signaling cascade, regulating the expression of various pro-inflammatory genes, including those encoding cytokines and chemokines [40]. This activation was assessed by stimulating the TLR4/MD-2 signaling pathway. This protocol was validated using LPS as the TLR4 ligand known for its agonistic effects. LPS stimulates TLR4/MD-2 signaling, activating the IKK complex and causing phosphorylation of IκB, a protein that inhibits NF-κB function [41]. Translocation of NF-κB into the nucleus leads to SEAP production. Therefore, SEAP secretion into the cell medium is correlated with the activation of these signaling pathways [42,43]. 

The screening results for agonist limonoids are shown in Figure S1, indicating that most of the compounds have no significant agonist activity and have no activity to activate the NF-κB pathway. However, compound **17** displayed a weak agonist effect compared to LPS (−)-treated cells. Furthermore, the antagonistic evaluation reveals that compounds **3**, **5**, **11**, **13**, and **17** have an inhibition effect on SEAP secretion (Figure 2). Moreover, some of the compounds exhibited cytotoxicity effects, and the effect was increased in the presence of 10 ng/mL LPS (Table 1), with only **13** and **15** maintaining high cell viability after treatment. This suggested that the antagonist effect could have resulted from the cytotoxicity of the compounds.

Subsequently, ELISA was performed to determine the ability of compounds **1**–**17** to inhibit the secretion of pro-inflammatory cytokines in LPS-induced THP-1 cells. LPS stimulates the inflammatory response, leading to the synthesis and release of TNF-α, which activates the NF-κB and MAPK pathways. These pathways generate the production and secretion of various inflammatory markers such as NO, TNF-α, prostaglandin E2 (PGE2), IL-1β, and IL-6, which are closely associated with inflammatory diseases [44,45,46]. Therefore, inhibiting LPS-induced overproduction of these pro-inflammatory cytokines is a promising strategy for developing anti-inflammatory agents.

As depicted in Figure 3, the relative TNF-α, IL-6, IL-1β, and MCP-1 levels significantly increased after LPS (10 ng/mL) treatment, reaching approximately 150-times fold for TNF-α, over 15-times fold for IL-6 and IL-1β, and approximately 30-times fold for MCP-1 compared to the LPS (−) control group (## *p* < 0.0001). Notably, compounds **3**–**5**, **9**, and **14**–**17** inhibited either IL-6, IL-1β, or MCP-1; meanwhile, the other compound showed no significant inhibition.

Further analysis of the selected compounds at concentrations of 1, 5, 10, and 20 µM revealed that compounds **3**–**5**, **9**, **12**, and **15** significantly inhibited TNF-α expression, displaying IC_50_ values ranging from 2.15 to 16.43 µM. Compounds **3**, **5**, **9**, **15**, and **16** potently inhibited IL-6 expression, with IC_50_ values ranging from 1.13 to 7.37 µM. Meanwhile, only compounds **3** and **5** inhibited LPS-induced MCP-1 expression, with IC_50_ values of 0.87 to 2.11 µM. Summarized in Table 2, the calculated selectivity index (SI) ratios (preferably ≥ 10) for the selected limonoid compounds were presented to indicate the selectivity and potential safety of the tested compounds [47]. Compound **5** demonstrated significant inhibition of pro-inflammatory cytokines. However, it also exhibited some level of cytotoxicity against THP-1 cells. Nonetheless, the inhibitory effect outweighs the cytotoxicity, particularly for the inhibition of MCP-1 expression, as evidenced by a selectivity index (SI) value of 16.76. Compound **9** showed an SI ratio of 49.92 and 94.98 for TNF-α and IL-6, respectively, suggesting its potential as a bioactive compound. Similarly, compounds **4**, **15**, and **16** demonstrated potential specificity, particularly for IL-6 inhibition. Additionally, all these compounds have been tested for their cytotoxic effect against RAW264.7 macrophage at concentrations of 5, 10, and 20 µM (Table S1), and compounds **4**, **9**, **15**, and **16** exhibited low-to-negligible cytotoxicity against RAW264.7 macrophage at a given concentration. In contrast, compound **5** demonstrated a dose-dependent cytotoxicity, indicating a less desirable property in drug discovery research, which raises concerns about the potential impact of compound **5**.

Structure–activity relationship elucidation of the limonoid compounds revealed that compounds **3**, **4**, and **9** were the most active inhibitors of either three or all four of the evaluated pro-inflammatory markers. These compounds share a common structural feature, a limonoid azadirone scaffold with an intact furan group. The only variation was in the hydroxy or acetoxy substituents at positions C-6 and C-7. Although this slight difference had a minor impact on their activity, the inhibitory effects remained remarkably strong. This suggests that the specific scaffold and positional arrangement of the hydroxy or acetoxy substituents play a role in the anti-inflammatory properties of these limonoids.

Numerous studies have demonstrated a strong correlation between the excessive production of pro-inflammatory cytokines and chemokines, such as TNF-α, IL-6, IL-1β, and MCP-1, and the development of inflammatory diseases. TNF-α, IL-6, and IL-1β play a critical role in various inflammatory diseases, while MCP-1 expression is correlated with the pathogenesis of certain diseases or viral infections [5,48,49]. Consequently, suppression of TNF-α, IL-6, IL-1β, and MCP-1 production could be an effective approach to inhibiting abnormal inflammatory responses. In the current study, compounds **4**, **9**, and **15**–**17** demonstrated remarkable selective inhibition towards two or three markers, while **3** and **5** exhibited extensive inhibition of TNF-α, IL-6, IL-1β, and MCP-1 expression in the LPS-stimulated THP-1 cell at a concentration below 20 µM. However, for further research, considering safety, efficacy, and clinical relevance is suggested.

In inflammation conditions, the p38 MAPK signaling pathway plays a critical role in various cellular processes. The p38 MAPK pathway plays a role as a central regulator of the expression and activity of pro-inflammatory cytokines, including TNF-α, IL-1, IL-2, IL-6, IL-7, and IL-8. This pathway involves various cell types, including macrophages and monocytes. Consequently, inhibition of the p38 MAPK pathway is regarded as an effective therapeutic strategy for treating inflammatory diseases. The suppression of p-p38 MAPK expression leads to a reduction in pro-inflammatory production and, subsequently, alleviation of inflammatory responses [50]. As demonstrated in this study, compounds **3**, **4**, **5**, **9**, **12**, **15**, and **16** significantly suppressed the expression of phosphorylated-p38 expression in THP-1 cells stimulated with LPS. These findings suggest that the inhibition effect of the MAPK signaling pathway caused by the limonoid treatment leads to the reduction in pro-inflammatory cytokines levels, such as TNF-α, IL-6, and IL-1β. Thus, these compounds may act as anti-inflammatory agents by modulating the MAPK pathway and subsequently decreasing the production of pro-inflammatory cytokines.

## 5. Conclusions

In conclusion, our preliminary in vitro investigation of the anti-inflammatory potential of limonoids from *Chisocheton* plants reveals promising results, with demonstration of significant inhibition of TNF-α, IL-6, IL-1β, and MCP-1 expressions in LPS-stimulated THP-1 cells. In particular, compounds **3**–**5**, **9**, and **14**–**16** resulted in IC_50_ values below 20 µM. Particularly, compound **9** demonstrated high selectivity for both TNF-α and IL-6, indicating its potential safety. Additionally, the evaluation of the MAPK signaling pathway suggests that compounds **4**, **9**, **12**, **15**, and **16** significantly suppressed the expression of phosphorylated-p38 MAPK in LPS-stimulated THP-1 cells. These findings indicate that the anti-inflammatory effects of the compounds may be due to their modulation of the MAPK pathway, resulting in the reduction in pro-inflammatory cytokines. This supports the use of limonoids from *Chisocheton* plants as promising candidates for anti-inflammatory agents.

## Figures and Tables

**Table 1:** Effects of 17 tested compounds on the cell viability.

Samples ^1^	Viability Cell (%)			
	HEK-Blue^TM^ hTLR4		THP-1	
	Without LPS	LPS-Induced (10 ng/mL)	Without LPS	LPS-Induced (10 ng/mL)
Control ^2^	100.00 ± 5.33	100.00 ± 9.41	100.00 ± 5.3	100.00 ± 4.20
**1**	>99.90	>99.90	>99.90	>99.90
**2**	61.20 ± 1.22	75.49 ± 13.78	>99.90	>99.90
**3**	20.74 ± 3.08	6.79 ± 6.22	>99.90	>99.90
**4**	>99.90	>99.90	>99.90	>99.90
**5**	43.29 ± 6.02	23.61 ± 2.61	66.73 ± 6.02	52.04 ± 6.51
**6**	77.06 ± 2.13	58.31 ± 1.89	90.69 ± 3.82	>99.90
**7**	92.92 ± 2.11	87.29 ± 4.00	>99.90	>99.90
**8**	>99.90	88.01 ± 6.37	>99.90	94.02 ± 9.84
**9**	>99.90	74.77 ± 6.22	>99.90	83.86 ± 7.60
**10**	>99.90	>99.90	>99.90	>99.90
**11**	52.61 ± 4.74	9.84 ± 7.34	>99.90	>99.90
**12**	>99.90	>99.90	>99.90	96.73 ± 4.30
**13**	92.41 ± 10.63	>99.90	90.75 ± 3.23	>99.90
**14**	>99.90	>99.90	>99.90	91.11 ± 9.03
**15**	>99.90	84.38 ± 18.67	>99.90	>99.90
**16**	>99.90	>99.90	90.40 ± 7.60	>99.90
**17**	>99.90	64.17 ± 9.05	87.27 ± 4.18	>99.90

^1^ Compound concentration; 20 µM against HEK-Blue^TM^ hTLR4 cells and 10 µM against THP-1 cells, ^2^ Control: 1% EtOH in medium.

**Table 2:** The summary of IC_50_ values of selected compounds and their selectivity index (SI) for evaluated pro-inflammatory marker.

Samples	THP-1	TNF-α		IL-6		MCP-1	
	CC_50_^1^ (µM)	IC_50_^2^ (µM)	SI ^3^	IC_50_^2^ (µM)	SI ^3^	IC_50_^2^ (µM)	SI ^3^
Dex	28.23	2.17	13.01	0.72	39.20	0.50	56.46
3	20.29	9.18	2.21	3.06	6.63	2.11	9.61
4	37.96	5.60	6.78	1.64	23.15	- *	- *
5	14.58	5.76	2.53	- *	- *	0.87	16.76
9	107.33	2.15	49.92	1.13	94.98	- *	- *
12	36.91	13.89	2.66	- *	- *	- *	- *
15	96.60	16.43	5.88	7.37	13.11	- *	- *
16	110.39	- *	- *	5.69	19.40	- *	- *

^1^ CC_50_ is the sample concentration required to cause 50% cytotoxicity against the cells. ^2^ IC_50_ is the concentration required to produce 50% inhibition of the evaluated pro-inflammatory marker. ^3^ SI (selectivity index, CC_50_/IC_50_). * Not determined.

## Data Availability

The supporting data for the findings presented in this study are available upon request from the corresponding authors.

## Supplementary Materials

The following supporting information can be downloaded at: https://www.mdpi.com/article/10.3390/cimb46010058/s1, Figure S1: Agonist effects of 17 Limonoid compounds (**1**–**17**) at a concentration of 20 µM on the NF-κB activation in HEK-Blue^TM^ hTLR4 Cells. All data from four independent experiments are expressed as mean ± SD. ## *p* < 0.0001 vs. culture medium-only control group; * *p* < 0.01, ** *p* < 0.001, *** *p* < 0.0001 vs. culture medium-only control group. Analyzed by one-way ANOVA, followed by Dunnett’s test performed using GraphPad Prism 9; Figure S2: Effects of dexamethasone and compounds **3**–**5**, **9**, **11**, **12**, and **15**–**17** in concentrations of 1, 5, 10, and 20 µM on the viability of THP-1 Cells presented as a heatmap. Figure S3. Western blot result of compounds **3**, **4**, **5**, **9**, **12**, **15**, and **16** on MAPK activation ((**A**) phosphorylated p38 MAPK; (**B**) total p38 MAPK) in LPS-stimulated THP-1 cells; Table S1: Cytotoxic effects of 17 tested compounds against RAW264.7 macrophage.
