# Supplementary material for: In Vitro Anti-Inflammatory Study of Limonoids Isolated from Chisocheton Plants
Source: Curr Issues Mol Biol. 2024 Jan 20;46(1):909–22. doi: 10.3390/cimb46010058 (PMC10814725; doi:10.3390/cimb46010058)
Supplement: Supplementary file 1 [file cimb-46-00058-s001.zip › cimb-2817368-supplementary.pdf]

Supplementary Files

In vitro Anti-inflammatory Study of Limonoids Isolated from *Chisocheton* Plants

Erina Hilmayanti[1,3], Xuhao Huang[1], Supriatno Salam[5], Nurlelasari[3], Unang Supratman[3,4], Kazuya Kabayama[1,2*], and Koichi Fukase [1,2,*]

[1] Department of Chemistry, Graduate School of Science, Osaka University, Toyonaka 560-0043, Japan
[2] Project Research Center for Fundamental Sciences, Osaka University, Toyonaka 560-0043, Japan
[3] Department of Chemistry, Faculty of Mathematics and Natural Sciences, Universitas Padjadjaran, Jatinangor 45363, Indonesia
[4] Central Laboratory, Universitas Padjadjaran, Jatinangor 45363, Indonesia
[5] Faculty of Pharmacy, Universitas Mulawarman, Samarinda, Kalimantan Timur 75123, Indonesia
* Correspondence: koichi@chem.sci.osaka-u.ac.jp; Tel.: +81-6-6850-5388 (K.F.); kaba@chem.sci.osaka-u.ac.jp; Tel.: +81-6-6850-5192 (K.K.)

Contents

Figure S1. Agonist effects of 17 Limonoid compounds (**1–17**) at a concentration of 20 μM on the NF-κB activation in HEK-BlueTM hTLR4 Cells. All data from four independent experiments are expressed as mean±SD. ##p<0.0001 vs. culture medium-only control group; *p<0.01, **p<0.001, ***p<0.0001 vs. culture medium-only control group. Analyzed by one-way ANOVA, followed by Dunnett's test using GraphPad Prism 9.

Figure S2. Effects of dexamethasone, and compounds **3-5**, **9, 11-12,** and **15-17** in concentrations of 1, 5, 10, and 20 μM on the viability of THP-1 Cells presented as a heatmap.

Figure S3. Western blot result of compounds **3, 4, 5, 9, 12, 15,** and **16** on MAPK activation (A: phosphorylated p38 MAPK; B: total p38 MAPK) in LPS-stimulated THP-1 cells.

Table S1. Cytotoxic effects of 17 tested compounds against RAW264.7 macrophage.

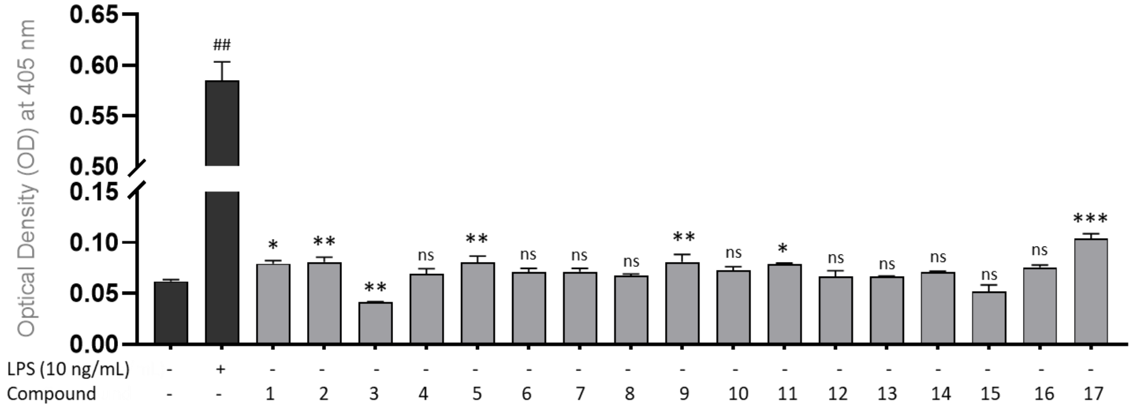

Figure S1. Agonist effects of 17 Limonoid compounds (**1–17**) at a concentration of 20 μM on the NF-κB activation in HEK-Blue™ hTLR4 Cells. All data from four independent experiments are expressed as mean±SD. ##p<0.0001 vs. culture medium-only control group; *p<0.01, **p<0.001, ***p<0.0001 vs. culture medium-only control group. Analyzed by one-way ANOVA, followed by Dunnett's test using GraphPad Prism 9.

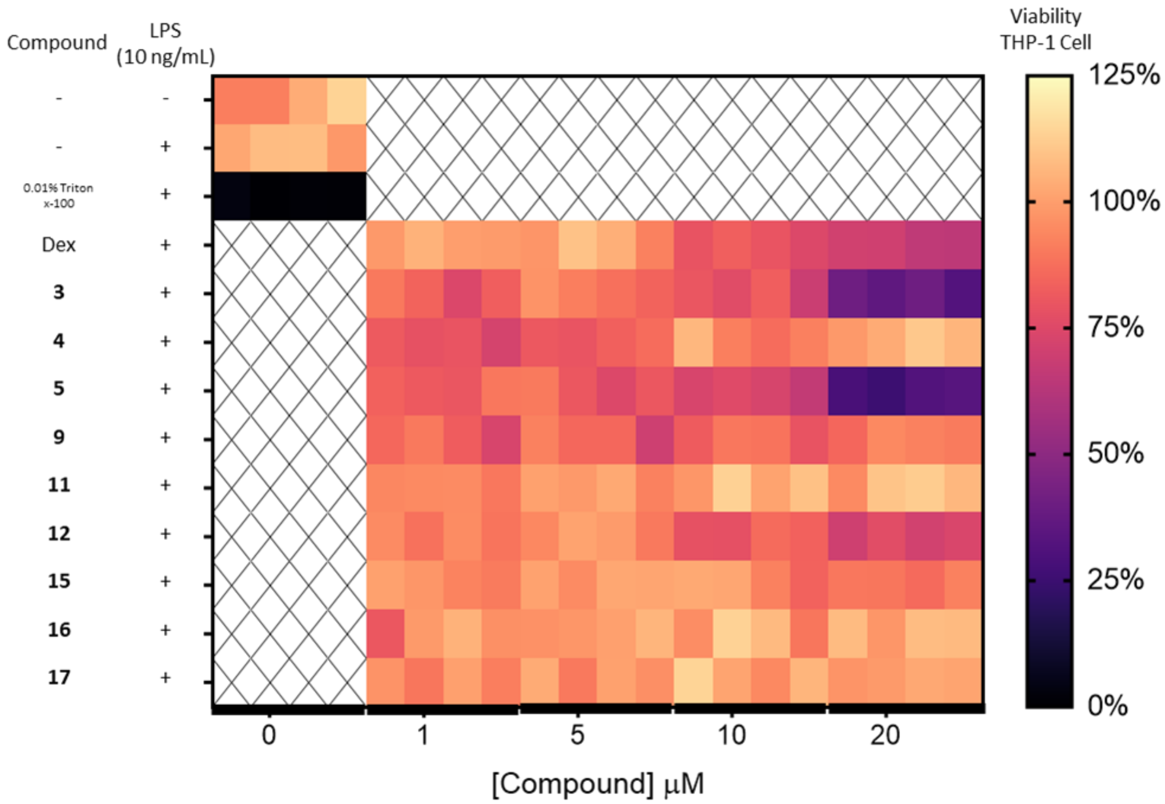

Figure S2. Effects of dexamethasone, and compounds **3**-**5**, **9, 11-12,** and **15-17** in concentrations of 1, 5, 10, and 20 μM on the viability of THP-1 Cells presented as a heatmap.

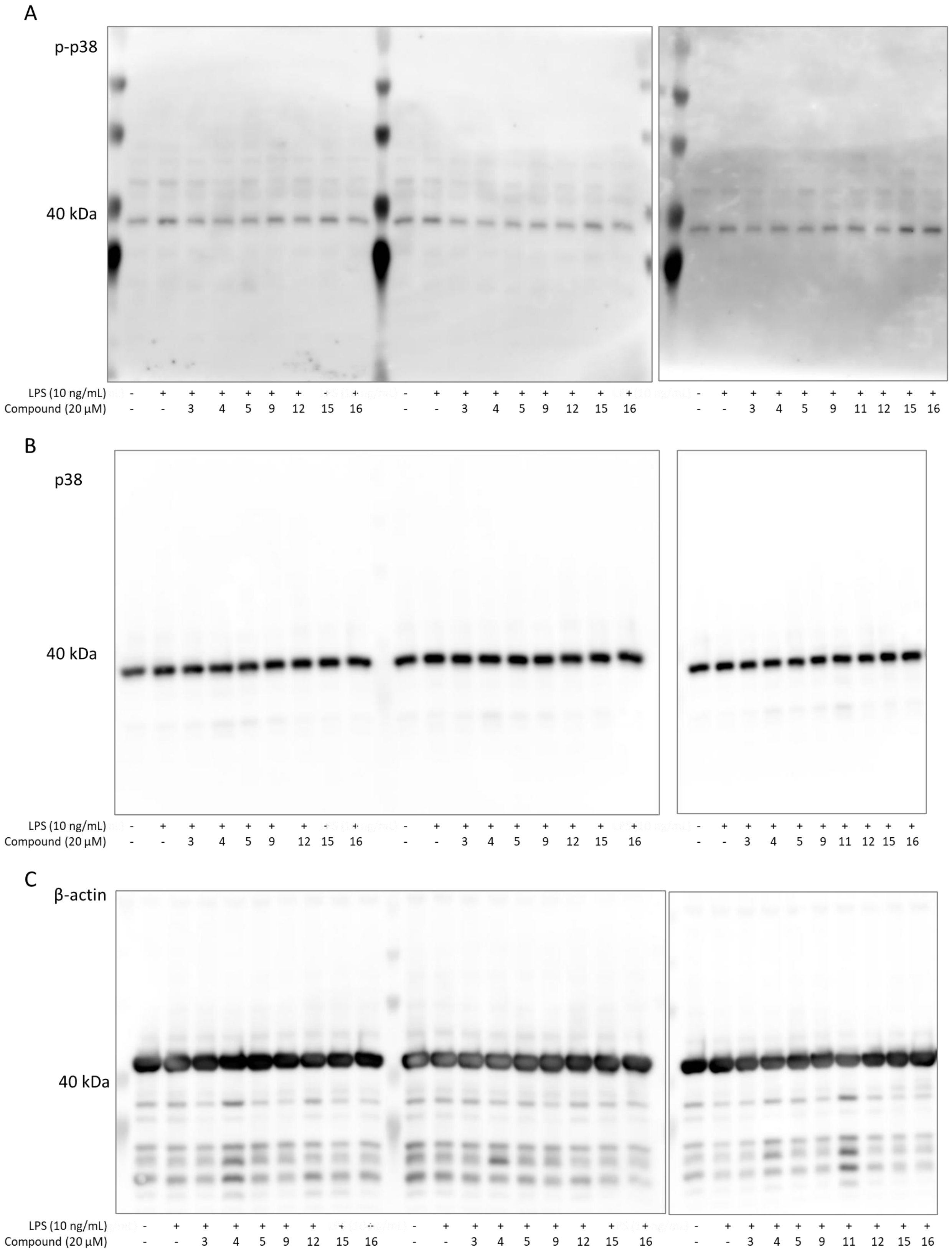

Figure S3. Western blot result of compounds **3, 4, 5, 9, 12, 15,** and **16** on p-p38, p38 MAPK activation and β-actin in LPS-stimulated THP-1 cells.

Table S1. Cytotoxic effects of 17 tested compounds against RAW264.7 macrophage.

Samples	Viability cell (%)		
	RAW 264.7		
	5 µM	10 µM	20 µM
Control*	>99.90	>99.90	>99.90
Etoposide	67.53 ± 2.36	54.79 ± 2.58	38.85 ± 3.19
**1**	>99.90	>99.90	>99.90
**2**	96.25 ± 3.67	>99.90	>99.90
**3**	90.27 ± 5.24	91.44 ± 8.26	70.21 ± 2.29
**4**	85.04 ± 5.66	85.92 ± 5.34	82.41 ± 1.15
**5**	83.58 ± 8.91	84.29 ± 0.28	3.19 ± 1.24
**6**	103.06 ± 6.05	>99.90	96.66 ± 2.14
**7**	>99.90	>99.90	95.88 ± 2.19
**8**	89.47 ± 9.38	94.75 ± 0.32	87.46 ± 3.33
**9**	93.76 ± 1.86	89.94 ± 2.44	79.41 ± 10.16
**10**	93.19 ± 4.20	82.06 ± 1.31	86.14 ± 4.07
**11**	90.10 ± 7.88	79.02 ± 1.58	78.88 ± 2.21
**12**	>99.90	>99.90	89.39 ± 6.90
**13**	>99.90	>99.90	91.89 ± 4.37
**14**	>99.90	88.19 ± 3.86	91.33 ± 4.19
**15**	92.82 ± 1.79	91.90 ± 3.59	90.91 ± 4.95
**16**	91.79 ± 3.18	81.32 ± 7.05	89.42 ± 7.08
**17**	77.49 ± 10.22	72.24 ± 4.68	73.09 ± 2.02

*Control: 1% EtOH in medium
